# The Clinical Profile of Severe Pediatric Malaria in an Area Targeted for Routine RTS,S/AS01 Malaria Vaccination in Western Kenya

**DOI:** 10.1093/cid/ciz844

**Published:** 2019-08-26

**Authors:** Samuel Akech, Mercy Chepkirui, Morris Ogero, Ambrose Agweyu, Grace Irimu, Mike English, Robert W Snow

**Affiliations:** 1 Kenya Medical Research Institute/Wellcome Trust Research Programme, Nairobi, Kenya; 2 Department of Paediatrics and Child Health, University of Nairobi, Nairobi, Kenya; 3 Centre for Tropical Medicine and Global Health, Nuffield Department of Clinical Medicine, University of Oxford, Oxford, United Kingdom

**Keywords:** malaria, severe, admissions, children, Kenya

## Abstract

**Background:**

The malaria prevalence has declined in western Kenya, resulting in the risk of neurological phenotypes in older children. This study investigates the clinical profile of pediatric malaria admissions ahead of the introduction of the RTS,S/AS01 vaccine.

**Methods:**

Malaria admissions in children aged 1 month to 15 years were identified from routine, standardized, inpatient clinical surveillance data collected between 2015 and 2018 from 4 hospitals in western Kenya. Malaria phenotypes were defined based on available data.

**Results:**

There were 5766 malaria admissions documented. The median age was 36 months (interquartile range, 18–60): 15% were aged between 1–11 months of age, 33% were aged 1–23 months of age, and 70% were aged 1 month to 5 years. At admission, 2340 (40.6%) children had severe malaria: 421/2208 (19.1%) had impaired consciousness, 665/2240 (29.7%) had an inability to drink or breastfeed, 317/2340 (13.6%) had experienced 2 or more convulsions, 1057/2340 (45.2%) had severe anemia, and 441/2239 (19.7%) had severe respiratory distress. Overall, 211 (3.7%) children admitted with malaria died; 163/211 (77% deaths, case fatality rate 7.0%) and 48/211 (23% deaths, case fatality rate 1.4%) met the criteria for severe malaria and nonsevere malaria at admission, respectively. The median age for fatal cases was 33 months (interquartile range, 12–72) and the case fatality rate was highest in those unconscious (44.4%).

**Conclusions:**

Severe malaria in western Kenya is still predominantly seen among the younger pediatric age group and current interventions targeted for those <5 years are appropriate. However, there are increasing numbers of children older than 5 years admitted with malaria, and ongoing hospital surveillance would identify when interventions should target older children.

**(See the Editorial Commentary by Castelli on pages 381–2.)**

A decline in the malaria parasite prevalence in western Kenya over the last 25 years has led to indications of a shift of severe malaria to older children, but malaria is still predominantly seen in the younger, pediatric age group.

There has been an unprecedented decline in the intensity of *Plasmodium falciparum* transmission in Africa since 2000 [1], resulting in a decline in the malaria burden across most countries [2]. These reductions have been attributed to a scaling up of vector control and improved case management [2]. However, these declines have stagnated in recent years [1, 2]; therefore, funding, the scope of coverage of existing interventions, and new interventions must increase to accelerate any future declines in transmission and the disease burden. As the landscape of malaria transmission changes, countries are being encouraged to tailor existing and novel sub-national strategic controls to more nuanced local epidemiology [3].

Over the last 25 years in Kenya, there has been an 88% overall reduction in the prevalence of malaria infection; however, 8 counties surrounding Lake Victoria remain highly endemic, despite a declining transmission since 2000 [4], and are the current focus of efforts to increase levels of vector control though the distribution of insecticide-treated nets, indoor residual house spraying, and larval source management [5, 6]. Following the World Health Organization (WHO) recommendations for the phased, monitored introduction of RTS,S/AS01 in 2016 [7, 8], Kenya plans to introduce RTS,S/AS01 into the highly endemic 8 counties in western Kenya in 2019 [9].

The clinical epidemiology of severe, life-threatening malaria was characterized during the 1990s [10] and its changing patterns have been described over 25 years along the Kenyan coast [11]. In western Kenya, during the early 1990s, the community-based malaria infection prevalence around the Siaya District hospital was over 80% [4, 10]. Under this level of transmission intensity, 75% of pediatric malaria hospitalizations were below 2 years of age, predominantly due to severe malaria anemia, with a few cases of cerebral malaria [10]. During the 1990s at Kilifi, on the Kenyan coast, the community prevalence of malaria infection was lower than in Siaya and malaria hospitalizations were among older children, with relatively more presentations with cerebral malaria [10]. As transmission intensity at Kilifi has declined over the last 25 years to very low levels, the mean age of malaria hospitalization has systematically increased [11, 12].

The intensity of transmission has declined in western Kenya since the 1990s [4]; however, there have been no detailed clinical descriptions of the patient ages, pathogenesis, and outcomes of pediatric malaria in this area since 1997. Here, we analyzed pediatric admission data assembled over 2 complete years since 2015 to provide a current understanding of the clinical epidemiology of malaria in an area poised to launch a scaling of existing and new vector-control strategies and RTS,S/AS01 vaccination among children aged 6 to 24 months.

## METHODS

### Study Sites

**Figure 1.**
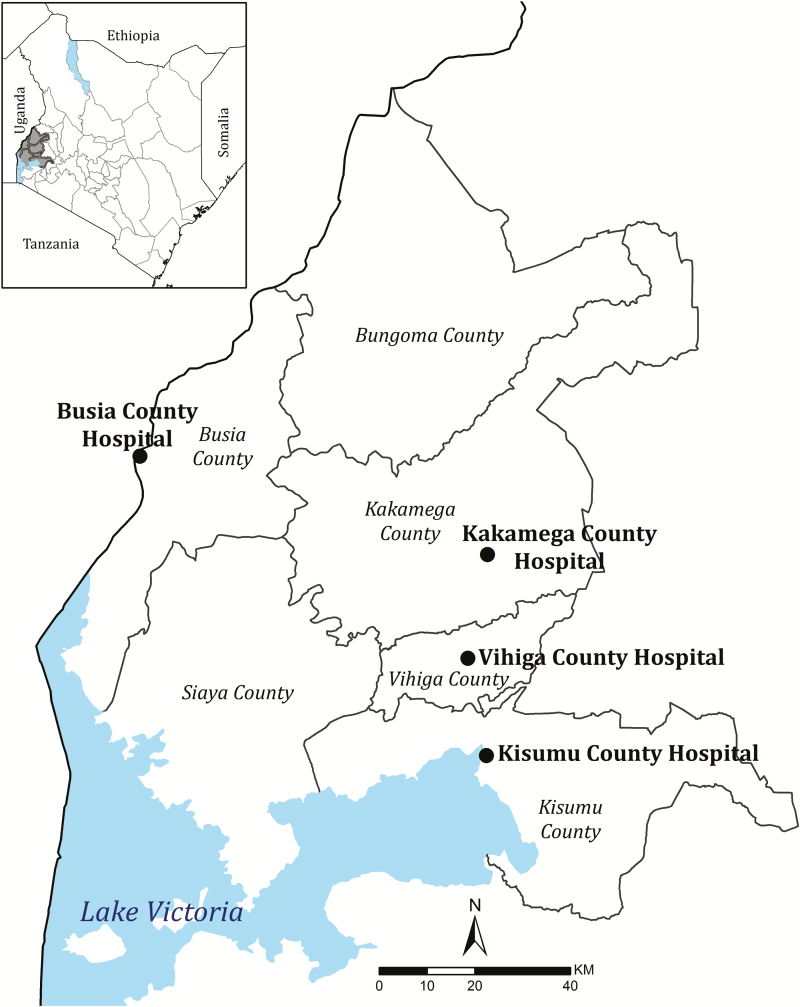
Map showing locations of study county hospitals in Western Kenya.

The present study was undertaken at 4 county referral–level hospitals located in western Kenya (Figure 1). The study is a secondary analysis of prospective data assembled as part of a system established in 2013 as part of a Clinical Information Network (CIN), as described previously [13, 14]. The 4 selected hospitals are in high–malaria transmission settings (parasite prevalences in children ≥30% in 2015 [4]) around Lake Victoria. The catchment areas to these hospitals form part of a wider area of similar malaria transmission intensity [4] that has been proposed for a pilot implementation of the RTS,S/AS01 malaria vaccine from 2019 through the existing routine immunization program, and are part of a multi-country postregistration study [8].

### Clinical Surveillance

The CIN surveillance system has been described elsewhere [13, 14]. In brief, slides for malaria microscopy and other tests are ordered by clinicians at the outpatient department prior to admission or from the ward. Patients who are admitted are further assessed by pediatric ward clinicians and observations are recorded using a standardized pediatric admission record. The record captures the patient’s history, vital signs, general clinical examinations of the airway, and respiratory, circulatory, and neurological systems. The neurological status is assessed using the alert, response to voice, response to pain, unconscious (AVPU) scale and the ability to drink or breastfeed, as appropriate for age. Anemia is clinically assessed by an examination for palmar pallor (recorded as either absent, mild, or severe). Respiratory distress is assessed by an examination of the chest for indrawing and/or deep, acidotic breathing. Information on laboratory tests ordered at admission and during hospitalization, treatments prescribed at admission, and final discharge information are also collected from medical notes and entered into the database. CIN does not support laboratory testing and any tests or results are based on a hospital’s capacity.

### Standard Management for Malaria Admissions

CIN supplies, promotes, and monitors use of Kenyan pediatric guidelines [15], which are an adapted version of WHO guidelines [16], in each hospital. These guidelines recommend that children diagnosed with severe malaria are managed with parenteral artesunate at 3 mg/kg of body weight for children weighing 20 kg or less, or 2.4 mg/kg of body weight for children weighing over 20 kg. Parenteral artesunate, which is widely available [17, 18], is recommended for at least 24 hours, with administration continued until the child has improved and is able to take a full course of oral, artemisinin-based combination therapy medication. The supportive therapy recommended for severe malaria includes the treatment of hypoglycemia with dextrose when the glucose level is <2.2 mmol/l; a blood transfusion for children with hemoglobin levels less than 4 g/dL or 4–5 g/dL with respiratory distress, although delays in transfusion may occur [19]; maintenance intravenous fluids for children with circulatory impairment or impaired consciousness (sometimes through nasogastric feeding for impaired consciousness); and oxygen therapy for children with hypoxia (pulse oximeter reading <90%) or severe respiratory distress. However, experience from CIN hospitals has shown poor compliance for glucose testing for severely ill children unable to feed [20], linked to challenges in the continuous supply of appropriate diagnostics and the variable use of pulse oximetry [13].

### Data Capture and Verification

All medical notes are reviewed by data clerks stationed at the hospital and entered each day into a database designed in Research Electronic Data Capture [21, 22] that includes logical range checks, together with local and server-level cleaning scripts [21, 22], and any clerical errors are reconciled within 7 days. Approximately 10 records are randomly sampled and reentered on site bimonthly for accuracy at each hospital [17].

### Laboratory Surveillance

Malaria slide results are recorded as positive or negative, with neither species identification nor parasite density. Hematology measurements (hemoglobin) are recorded when requested; however, children may receive blood transfusions based on a clinical diagnosis of severe anemia or a documented severe pallor. Blood glucose, creatinine, lactate, bilirubin, and blood gas tests are rarely performed, and microbiology remains unreliable at all the hospital settings [13, 20].

### Case Definitions

Malaria was defined based upon a reanalysis of characteristics documented in medical notes at admission, in-patient notes, and information recorded at discharge. A malaria diagnosis was classified as (1) the presence of fever (defined as a history of fever or an axillary temperature ≥ 37.5°C) plus a positive malaria slide and a primary discharge diagnosis of malaria; (2) the presence of fever plus a primary clinical discharge diagnosis of malaria where results of a malaria test were not documented; or (3) if both a history of fever and a temperature were not recorded at admission but the patient had a positive malaria slide and a primary discharge diagnosis of malaria (Supplementary Figure 1).

It was not possible to define the broad classification of severe malaria according to the WHO definition [23, 24], which includes hypoglycemia, hyperlactatemia, hemoglobinuria, hyperparasitemia, radiological pulmonary edema, shock, jaundice (hyperbilirubinemia), and renal failure. The focus is, therefore, on common clinical syndromes of severe malaria in African children [25], for which information was available to define signs denoting cerebral involvement (AVPU), 2 or more convulsions, measures or signs of severe anemia, and respiratory distress (Table 1).

**Table 1.** Case Definitions for Severe Malaria Syndromes

	WHO Definition [1, 2]	Definition Used in Present Study
**Impaired consciousness**	Blantyre coma score <3 in children; cerebral malaria is defined as a coma persisting for >30 minutes after a seizure	2 definitions of cerebral malaria used: cerebral malaria 1 was defined as being unconscious, as measured on the AVPU scale (AVPU score = U); cerebral malaria 2 was defined as a child responding only to pain or being unconscious (AVPU score = U or P); children with AVPU score = V were also considered to have severe malaria
**Prostration**	Inability to sit if normally able to sit or inability to breastfeed if previously not sitting	Inability to drink or breastfeed but fully alert (AVPU score = A) used as proxy for prostration
**Acidosis, respiratory distress**	A base deficit of >8 meq/l, plasma bicarbonate of <15 mM, venous plasma lactate >5 mM, or severe acidosis manifesting clinically as respiratory distress, defined as rapid, deep, and labored breathing	Chest indrawing or deep/acidotic breathing on examination
**Severe malarial anemia**	A hemoglobin concentration <5 g/dl or a hematocrit of <15% in children <12 years of age, together with a parasite count >10 000/µl	Defined as hemoglobin ≤5 g/dl in the presence of any malaria parasitemia (parasite count not done). If the hemoglobin level was not documented, then severe anemia was also defined as any of the following: clinical diagnosis of severe anemia, documented severe palmar pallor, or transfusion given or prescribed
**Multiple convulsions**	More than 2 episodes within 24 hours	Same definition

Abbreviations: A, alert; AVPU, alert, response to voice, response to pain, unconscious scale; P, responsive to pain; U, unconscious; V, responsive to a voice; WHO, World Health Organization.

Data were analyzed for 2 complete years on either side of a national health worker strike, from December 2015 to November 2016 and from November 2017 to October 2018 [26]. The analysis was in all pediatric admissions aged between 1 month to 15 years.

## RESULTS

### Malaria Admissions

A total of 14 999 children aged <15 years were admitted to the 4 hospitals over the 24 months of surveillance between 1 December 2015 and 31 October 2018 (Supplementary Figure). In brief, complete data on a measured temperature and/or history of fever was available for 82% of all presentations. Of those with fever documentation, a blood film result for malaria microscopy was available at presentation or during admission among 7919/10 345 (76.5%) patients, of which 4445 (56.1%) were positive for malaria parasites. Among the 2725 patients admitted without a recorded history of fever or axillary temperature, 863 had a blood slide taken, of whom 332 were positive. Among 2423 patients with a recorded fever but without a blood slide result, 989 had a final discharge diagnosis of malaria. In summary, we treated 5766 patients (38.4% of all admissions aged 1 month to 15 years) as having a primary diagnosis of malaria, based predominately (82.8%) on a combination of fever, the presence of malaria parasites, and a final discharge diagnosis of malaria (Supplementary Figure). Information on age, discharge diagnosis, and outcome were available for all malaria-defined admissions.

### Severe Malaria Classifications

Among 5766 malaria admissions aged <15 years, complete records were available for 5219 (90.5%) on the AVPU score, 5147 (89.3%) on the ability to drink/breastfeed, and 5308 (92.1%) on the history of convulsions. Convulsions were reported in 1650 children and the number of convulsions were recorded in 1292 (78.3% of those with a history of convulsions). Hemoglobin results were available for 2085 (36.2%) of malaria admissions and, where this was not available, information on an assessment for severe pallor (5272, 91%) or whether they received a blood transfusion (5450, 95%) was available to identify those patients with severe anemia. Documented evidence of indrawing and/or deep breathing was available for 5307 (92.0%) malaria admissions. A total of 2340 (40.6%) children admitted with malaria had severe malaria, defined by the presence of any criteria in Table 1.

**Figure 2.**
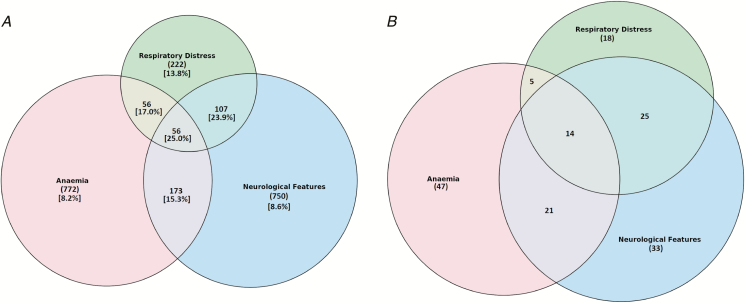
*A*, Overlap of malaria clinical syndromes in all malaria admissions (n = 5766), n represents total number of admissions with malaria. Square brackets denote percentage case fatality rates. *B*, Overlap of malaria clinical syndromes in malaria deaths (n = 211), n represents the total number of deaths in children admitted with malaria.

Among malaria admissions, the strict definition of cerebral malaria (AVPU score = U, cerebral malaria 1) was present in 81 (1.6%) of children at admission. A wider definition of cerebral involvement (AVPU score = P or U, cerebral malaria 2) was present in 299 (5.7%) children. There were 122 (2.3%) regarded as conscious but with an inappropriate response to voice (AVPU score = V). The inability to drink/breastfeed was present in 665 (12.6%) of children at presentation to the pediatric ward (Table 2). Among all malaria admissions, 1057 (18.3%) had severe anemia, of whom 576 had hemoglobin levels ≤5 g/dL and 481 had severe anemia identified using clinical criteria (severe pallor or blood transfusion). Severe respiratory distress was present in 441 (8.3%) malaria admissions: 92 had deep breathing only, 305 had chest indrawing only, and 44 had both deep breathing and chest indrawing. The distributions of the various clinical phenotypes in severe malaria are summarized in Figure 2A.

**Table 2.** Malaria Admissions and the Severe Disease Syndromes

Severity feature	n (%) or n/N (%)	Median age, months (IQR)	Case fatality, (%, 95% CI)
All malaria admissions	5766	36 (18–60)	211/5766 (3.7, 3.2–4.2)
Uncomplicated malaria	3426	36 (18–66)	48/3426 (1.4, 1.0–1.8)
Severe malaria	2340	36 (16–60)	163/2340 (7.0, 6.0–8.1)
Severe malaria admissions			
AVPU score = U; cerebral malaria 1	81/2208 (3.7)	48 (30–84)	36/81 (44.4, 33.4–55.9)
AVPU score = U or P; cerebral malaria 2	299/2208 (13.5)	44 (20–72)	62/299 (20.7, 16.3–25.8)
AVPU score = V	122/2208 (5.5)	36 (24–63)	9/122 (7.4, 3.4–13.5)
Inability to drink/breastfeed	665/2240 (29.7)	32 (16–54)	22/665 (3.3, 2.1–5.0)
Convulsions ≥2	317/2340 (13.6)	30 (17–48)	12/317 (3.8, 2.0–6.5)
Anemia			
Severe anemia: clinical criteria or Hb ≤5 g/dL	1057/2340 (45.2)	36 (18–62)	87/1057 (8.2, 6.6–10.1)
Hemoglobin ≤5 g/dL	576/2340 (24.6)	36 (19–66)	33/576 (5.7, 4.0–8.0)
Clinically diagnosed severe anemia: Hb not documented	481/2340 (20.6)	36 (16–60)	54/481 (11.2, 8.5–14.3)
Respiratory symptoms			
Severe respiratory distress: deep breathing or chest indrawing	441/2239 (19.7)	19 (9–41)	61/441 (13.8, 10/7–17.4)
Deep breathing, no indrawing	136/2239 (6.1)	30 (16–51)	28/136 (20.6, 14.1–28.4)
Chest indrawing with or without deep breathing	349/2239 (15.6)	15 (9–36)	44/349 (12.6, 9.3–16.6)

Denomitators less than 2340 show cases with complete data; children may have had more than 1 severity feature.

Abbreviations: AVPU, alert, response to voice, response to pain, unconscious scale; CI, confidence interval; Hb, hemoglobin; IQR, interquartile range; P, responsive to pain; U, unconscious; V, responsive to a voice.

### Mortality in Children With Various Severe Malaria Clinical Syndromes

Overall, 211 (3.7%) children admitted with malaria died during hospitalization and 163 deaths occurred in children with severe malaria (case fatality rate 7.0%). The median age for fatal cases was 33 months (interquartile range [IQR] 12–72) and surviving children were of a similar age (median 36 months, IQR 18–60).

Case fatality rates were highest among unconscious children (AVPU score = U; 44.4%); however, fatality rates were also high for children who had an AVPU score of either P or U (20.7%) or of V (7.4%; Table 2). The case fatality rate in those with an inability to drink/breastfeed was much lower (3.3%). The case fatality rate for 317 children with 2 or more convulsions at admission was 3.8%. The case fatality rate for the composite definition of severe anemia was 8.2%, with rates of 5.7% in those with hemoglobin levels ≤5 g/dL and 11.2% in cases of clinically defined severe anemia without a hemoglobin measurement. The overall case fatality rate for those with severe respiratory distress was 13.8%, and this was higher in those with deep breathing (20.6%), compared to those with chest indrawing (12.6%; Table 2).

The mortality rate in children with severe anemia plus impaired consciousness (AVPU < alert or inability drink/breastfeed) was 15.3% (n = 35); it was 17.0% (n = 19) in those with severe anemia plus severe respiratory distress, 23.9% (n = 39) in those with impaired consciousness plus respiratory distress, and was highest, 25% (n = 14), in those children with a combination of severe anemia, severe respiratory distress, and impaired consciousness (Figure 2B). Otherwise, all 3 phenotypes were relatively common among the deaths (Figure 2B).

There were 48 deaths in children who did not have any of the severity features shown in Table 2 at admission, representing 23% of all the hospital deaths from malaria, but there was a case fatality rate of only 1.4% among children classified as having nonsevere malaria at admission. These deaths were in children without obvious comorbidities (data not shown) and 90% (43/48) had positive malaria slides, but they were older (median 48 months, IQR 14–98) compared to survivors (median 36 months, IQR 18–66) who had the same clinical status at admission. The mortality rate overall was higher among children aged 1 to 3 months (10.0%, 9/90), despite representing a small group of admissions, compared to the overall mortality rate amongst older children (3.6%). All 9 children aged 1 to 3 months who died had a positive malaria smear.

### Age Distribution of Malaria Admissions and Clinical Syndromes

**Figure 3.**
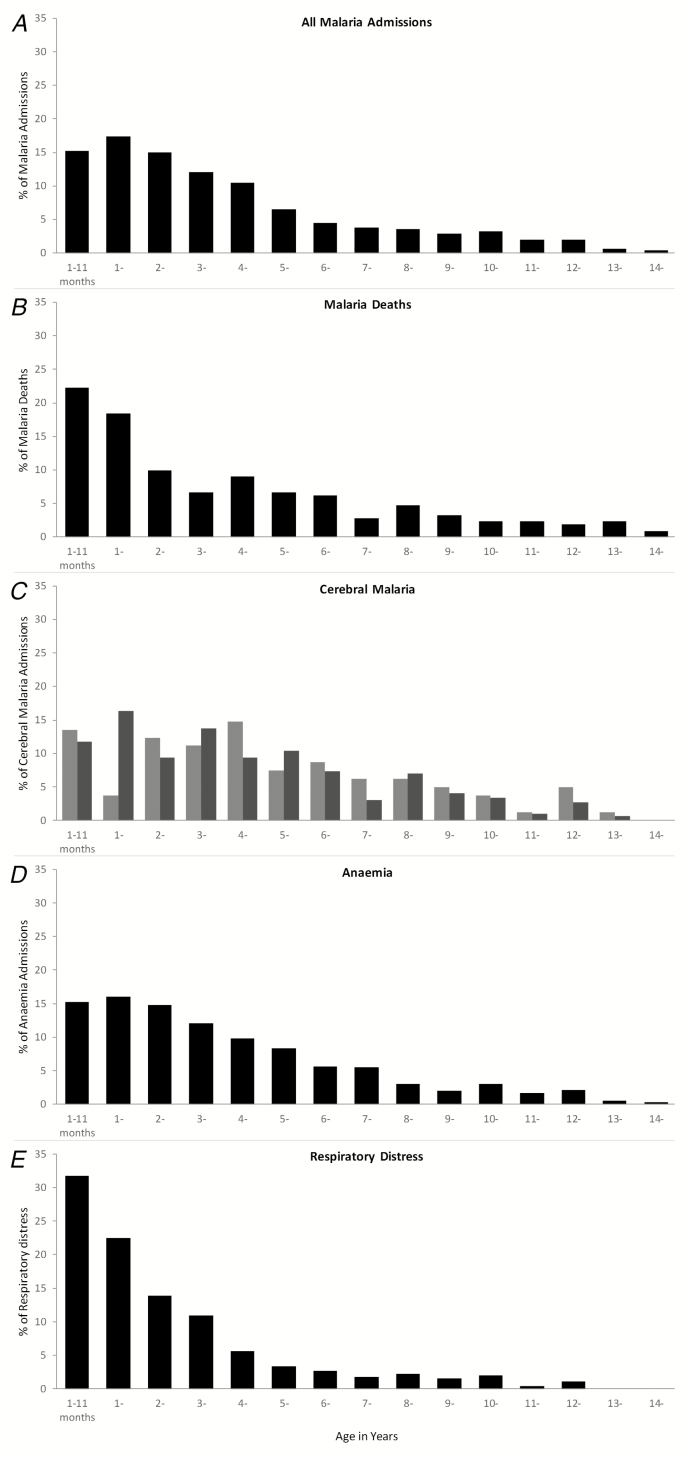
Percentage age distributions among children aged 1 month to 15 years for (*A*) 5766 malaria admissions, (*B*) 211 malaria deaths; (*C*) 81 cerebral malaria case definition 1 (AVPU score = U; light gray) and 299 cerebral malaria case definition 2 (AVPU score = P or U; dark gray); (*D*) 1057 severe malaria anemia cases; and (*E*) 414 malaria cases with respiratory distress. Abbreviations: AVPU, alert, response to voice, response to pain, unconscious scale; P, responsive to pain; U, unconscious.

The median age for all malaria admissions was 36 months (IQR 18–60). Of all malaria admissions to the pediatric ward, 15% were aged between 1–11 months of age, 33% were aged 1–23 months of age, and 70% were aged 1 month to 5 years (Figure 3A). Only 1.6% (90/5766) of malaria admissions were aged less than 3 months, and 82% had a positive malaria smear. Of the 211 deaths that occurred, 22% were in children aged 1–11 months, 41% were in children aged 1–23 months, and 66% were in children aged between 1 month and 5 years (Figure 3B). Admissions and deaths in children aged above 10 years were rare, comprising 489 (10%) of malaria admissions and 21 (10%) of malaria deaths. Children defined as having cerebral malaria based on an AVPU score of U were, on average, older than those with an AVPU score of P or U (Table 1; Figure 3C). Children who had a composite definition of severe malaria anemia (Figure 3D) or respiratory distress (Figure 3E) were younger on average than children with cerebral involvement (Table 1).

## DISCUSSION

Pediatric malaria and severe malaria hospitalizations in western Kenya still predominantly affect younger pediatric age groups (Figure 3A); however, the mean age at admission has shifted toward older children, when compared to hospital data from a neighboring hospital from the 1990s [10]. There are increasing numbers of children presenting at older age groups, including 30% who present above 5 years of age (Figure 3A), and an increasing proportion of admissions with cerebral involvement (Figure 3C). Comparisons between hospital settings 20 years ago suggested that as the intensity of transmission declined, functional immunity to severe malaria would be acquired later in childhood, resulting in risks among older children with different disease phenotypes [10, 27–31]. Subsequent studies of the long-term follow-up hospital surveillance of age and clinical phenotype changes against changing malaria transmission have been few. Where these studies have taken place, confirmation of a shifting age pattern toward older children has been documented when transmission declines [11, 12, 32, 33].

The case fatality rates for severe malaria remain high, despite the adoption of improved case-management guidelines in these hospitals [15, 17, 18, 20] (Table 2; Figure 3). Of malaria deaths, 48 (23%) did not have any of the prognostic characteristics of severe malaria on admission, and these deaths tended to occur among older children, compared to their surviving counterparts. It is possible that these children had some of the extended features of severe malaria not captured in our surveillance (previously identified as prognostic factors), progressed to have signs of severe malaria during admission, or had another condition unidentified because of limited diagnostics. Consistent with other site-specific studies [34, 35] and clinical trials [36] since the early 1990s [25], cerebral malaria, other neurological complications, severe anemia, and respiratory distress in this area of Kenya continue to have high case fatality rates, with increasing probabilities of death as symptoms overlap (Table 2; Figure 2). In our series, only 36% of malaria admissions had hemoglobin measured. The group we clinically defined as having severe anemia (severe pallor and/or blood transfusion) in the absence of a hemoglobin measurement had a higher mortality rate, compared to those where an admission hemoglobin measurement was used to define severe anemia (Table 2). In previous clinical observations of severe malaria, case fatality rates of severe malaria anemia have been lower than those of other syndromes [25]. This was true of our group, with a recorded hemoglobin level of <5 g/dl; however, those with a clinical definition may have other complications leading to high fatality rates, as previously described [37].

Preventing severe disease progression and improving timely access to emergency care must remain a priority across all settings in Africa. Access to hospital care in the area studied here is comparatively better than other areas of Kenya [38]; however, there remain unmet needs in managing severely ill patients with malaria in Africa [39]. As disease presentations become increasingly complex, with declining malaria transmission, improvements in supportive and primary treatments for severe malaria are still required [34, 40, 41].

Hospital surveillance of severe malaria and its outcomes provide important insights into the age profiles of life-threatening diseases in the surrounding communities, enabling policy-makers to redesign disease prevention targets. Despite a reduction in malaria transmission in western Kenya and a coincidental increase in the mean ages of patients with severe malaria, hospitalization, and deaths in hospital, malaria continues to be concentrated in children aged less than 36 months (60%; Figure 3A). The use of intermittent presumptive treatment in infancy [42] with effective drugs would provide an additional strategy to existing vector-control approaches in this area. Similarly, the RTS,S/AS01 vaccine will provide preerythrocytic immunity, with clinical protection for children up to 5 years of age [43], and this may have the effect of reducing immunity to blood-stage parasites, resulting in prolonged vulnerability and increased incidences of neurological phenotypes in older children [43, 44]. Both intermittent presumptive treatment in infancy and vaccination are strategies delivered through the routine, expanded program on immunization (EPI) services. It is, however, possible that transmission will change further in western Kenya following expanded vector control, and it might be anticipated that the disease burden will shift toward an age group not covered by EPI, in which case additional tools will need consideration. Presently, 30%, 29%, and 33% of the overall malaria hospital disease burden, severe disease burden, and malaria deaths, respectively, occur among children aged ≥5 years.

The surveillance system described here has been developed in partnership with the national and county-level ministries of health [13, 20]. The system involves training of staff, simplified electronic data capture tools, and quality assurance and feedback. It has not been designed specifically for malaria surveillance; as such, there are characteristics of severe malaria we were unable to describe, including hypoglycemia, hyperlactatemia, hyperbilirubinemia, and hyperparasitemia. It is possible, therefore, that we misclassified febrile syndromes as caused by malaria in children with incidental parasitemia, especially in settings with limited diagnostic capabilities. With improvement in point-of-care tests [45–48], future severe malaria surveillance can be improved. As part of the proposed evaluation of the RTS,S/AS01 in Kenya, pediatric surveillance will include modifications to existing tools to improve our ability to compare coma scoring, document hemoglobinuria, provide pathogen diagnoses of meningitis, and provide improved coverage of all clinical and hematological examinations among febrile presentations. This will enable a description of any changes in the clinical epidemiology of malaria and severe disease following the introduction of the vaccine. Hospitals provide unique settings to understanding the changing clinical epidemiology of pediatric infectious diseases and vaccine-preventable disease surveillance [49–54].

## CONCLUSION

In an area of western Kenya where the RTS,S/AS01 malaria vaccination is proposed as part of routine EPI, malaria admissions, severe malaria, and hospital malaria deaths continue to predominantly affect the younger pediatric age group. Case fatality rates are high among patients with cerebral malaria, which was an infrequent presentation in this part of Kenya 25 years ago. Neurological complications may increase with declining malaria transmission and may involve increasingly older patients outside of the EPI protection range. Hospital surveillance provides a routine, sustainable means to track the severe malaria phenotype, as well as future impacts of community-based controls.

## Supplementary Data

Supplementary materials are available at *Clinical Infectious Diseases* online. Consisting of data provided by the authors to benefit the reader, the posted materials are not copyedited and are the sole responsibility of the authors, so questions or comments should be addressed to the corresponding author.

ciz844_suppl_Supplementary_Figure

## Notes

***Author contributions.*** S. A. and R. W. S. designed the experiment and concept. M. C., M. O., and S. A. undertook the data cleaning and management and conducted the preliminary analysis. S. A. and R. W. S. reviewed the final data and analysis. S. A., M. E., and R. W. S. drafted the manuscript. All authors contributed to and approved the final version.

***Acknowledgments.*** The authors thank the Ministry of Health, which gave permission for this work to be developed and has supported the implementation of the Clinical Information Network (CIN); the county health executives, hospital management teams, Kenya Paediatric Association, Kenya Ministry of Health, and University of Nairobi for promoting the aims of the CIN; teams based in Vihiga (Victor Juma), Kakamega (Nick Aduro, Boniface Nyumbile, and Roselyne Malangachi), Busia (Emma Sarah Namulala) and Kisumu (Magdalene Kuria); members of the Kenya Medical Research Institute-Wellcome Trust program (David Kyalo, Abraham Lagat, Cynthia Khazenzi, and Basil Okola) for technical data assistance; and Philip Bejon and Kathryn Maitland for comments on an earlier version of the manuscript.

***Disclaimer.*** This work is published with the permission of the Director of the Kenya Medical Research Institute (KEMRI). The KEMRI Scientific and Ethical Review Unit (SERU) approved the CIN study (SERU #2465 and #3459). Data for this report are under the primary jurisdiction of the Ministry of Health in Kenya. Enquiries about using the data can be made to the KEMRI-Wellcome Trust Research Programme Data Governance Committee. The funders had no role in drafting or submitting this manuscript.

***Financial support.*** This work was supported by a Senior Wellcome Fellow award (number 207522 to M. E.) that provided funding to manage the CIN surveillance system and by the Wellcome Trust (grant numbers 092654 and 203077 to the Kenya Major Overseas Programme). S. A. is supported by the Initiative to Develop African Research Leaders Wellcome Trust (award number 107769). M. E. is supported as a Senior Wellcome Fellow (award number 207522). R. W. S. is funded as a Principal Wellcome Fellow (award numbers 103602 and 212176).

***Potential conflicts of interest.*** All other authors report no potential conflicts. All authors have submitted the ICMJE Form for Disclosure of Potential Conflicts of Interest. Conflicts that the editors consider relevant to the content of the manuscript have been disclosed.
