# Supplementary material for: The Clinical Profile of Severe Pediatric Malaria in an Area Targeted for Routine RTS,S/AS01 Malaria Vaccination in Western Kenya
Source: Clin Infect Dis. 2019 Aug 26;71(2):372–80. doi: 10.1093/cid/ciz844 (PMC7353324; doi:10.1093/cid/ciz844)
Supplement: ciz844_suppl_Supplementary_Figure [file ciz844_suppl_supplementary_figure.docx]

**Supplementary Figure: Flow chart showing identification of admissions with malaria -malaria cases (a) to (d)-shaded boxes**

Total admissions (N=15,022

Eligible (N=14,999)

Temperature ≥37.5°C or History of fever

(n=10,345)

Both temperature and history of fever not documented (n= 2,725)

Age >15 years=23

No history of fever or temperature <37.5°C (n=1,759)

Eligible admissions (n=14,829)

Clinical diagnosis of malaria as secondary diagnosis (n=170)

Slide ordered (n=9,371=91%)

Slide not ordered (n=971=9%)

Slide ordered (n=863=32%)

Slide not ordered (n=1,862=68%)

Slide positive

(n=4,445)

(a)

Slide negative

(n=3,474)

Clinical diagnosis of malaria (n=234)

(c)

Slide positive (n=332)

(d)

Slide negative (n=427)

Slide results missing (n=104)

Clinical diagnosis of malaria (n=43)

Slide results missing

(n=1,452)

Clinical diagnosis of malaria (n=755)

(b)

Clinical diagnosis of malaria (n=46)
